# Which States Matter? An Application of an Intelligent Discretization Method to Solve a Continuous POMDP in Conservation Biology

**DOI:** 10.1371/journal.pone.0028993

**Published:** 2012-02-17

**Authors:** Sam Nicol, Iadine Chadès

**Affiliations:** 1 Institute of Arctic Biology, University of Alaska Fairbanks, Fairbanks, Alaska, United States of America; 2 CSIRO Ecosystem Sciences, CSIRO, Brisbane, Queensland, Australia; University of Alberta, Canada

## Abstract

When managing populations of threatened species, conservation managers seek to make the best conservation decisions to avoid extinction. Making the best decision is difficult because the true population size and the effects of management are uncertain. Managers must allocate limited resources between actively protecting the species and monitoring. Resources spent on monitoring reduce expenditure on management that could be used to directly improve species persistence. However monitoring may prevent sub-optimal management actions being taken as a result of observation error. Partially observable Markov decision processes (POMDPs) can optimize management for populations with partial detectability, but the solution methods can only be applied when there are few discrete states. We use the Continuous U-Tree (CU-Tree) algorithm to discretely represent a continuous state space by using only the states that are necessary to maintain an optimal management policy. We exploit the compact discretization created by CU-Tree to solve a POMDP on the original continuous state space. We apply our method to a population of sea otters and explore the trade-off between allocating resources to management and monitoring. We show that accurately discovering the population size is less important than management for the long term survival of our otter population.

## Introduction

Conservation managers must manage populations of endangered species despite being uncertain about the population dynamics and the exact size of the population of interest. Resources spent on intensive monitoring reduce expenditure on management that directly improves species persistence. However monitoring may prevent poor management actions being taken due to observation error in abundance data. Partially observable Markov decision processes (POMDPs) are decision models that account for stochastic population dynamics as well as imperfect detection of the population. POMDPs are notoriously difficult to solve when the number of states is even moderately large because the solution algorithm efficiency decays exponentially with the size of the state space (finite-horizon problems have a complexity that is P-SPACE complete [1]; for infinite horizon problems the complexity is undecideable [2]). In recent years, efficient methods have been developed in artificial intelligence to solve POMDPs approximately providing a suite of tools that can be used in ecology and conservation biology. The methods to solve POMDPs require populations of individuals to be split into discrete states, but choosing a discretization introduces a dilemma: if there are too many states in the discretization, then the POMDP solution algorithm becomes intractable; but if too few states are chosen, then the discretization is too coarse and the results can lose their biological significance. Our paper's answer to the dilemma is to have just enough states so that managers can adequately discern between the effects of management actions.

Many ecological studies use continuous models (e.g. theta-logistic model, Beverton-Holt model [3]) to estimate population size. These models have the advantage of having well-known solutions and established methods for parameter estimation. However these models do not naturally suggest a state space discretization which is required for many optimal decision making techniques. In this paper we make use of an automated state space discretization method named Continuous U-Tree (CU-Tree) [4]. CU-Tree compactly discretizes the state space by finding the areas of the state space that are necessary to discern between the effects of the management actions. CU-Tree only adds states where they are statistically necessary to maintain the optimal solution, which can reduce the number of states without simplifying the biological dynamics. We exploit this property to find an optimal management policy for a partially observable population of the Washington sea otter.

### Case study: The Washington sea otter

We demonstrate the benefits of an intelligent discretization approach in conservation by designing an optimal management policy for a partially observable population of the Washington sea otter (*Enhydra lutris*). The Washington sea otter population is relatively small in geographic area and is located close to busy shipping lanes, meaning oil spills due to shipping accidents pose a considerable threat to the population. The population is partially observable, meaning that it is not possible to count the true population size exactly. The management objective is to maintain the sea otter population within 20% of the population carrying capacity given our belief in the accuracy of our population census (see methods section for further discussion of the management objective and the model).

We allow for four possible management actions to be taken at any time step. Only one action can be taken during each (annual) time step. We assume that a management agency can: (i) reduce the frequency of oil spills, for example by restricting shipping near sea otter habitat; (ii) reduce the damage caused by an oil spill; (iii) re-introduce otters, for example otters can be introduced from healthy populations in other parts of North America, or (iv) do nothing. When considering the partially observable problem, we also allow for a monitoring action, which improves our ability to detect the true population size.

We use the best-fit logistic growth (Ricker) model obtained by [5] (hereafter the ‘Gerber’ model) to determine the optimal state-based management actions to take under different oil spill scenarios. We then consider how imperfect detection during a population census will affect the results.

The paper is organized as follows: we first present the results of applying our new method to the sea otter problem. We then discuss the discretization method and POMDP methods in the context of solving complex optimization problems in conservation biology. In the final section we give further details of our methods including the ecological problem, continuous population model, the state space discretization algorithm and POMDP.

## Results

**Figure 1:**
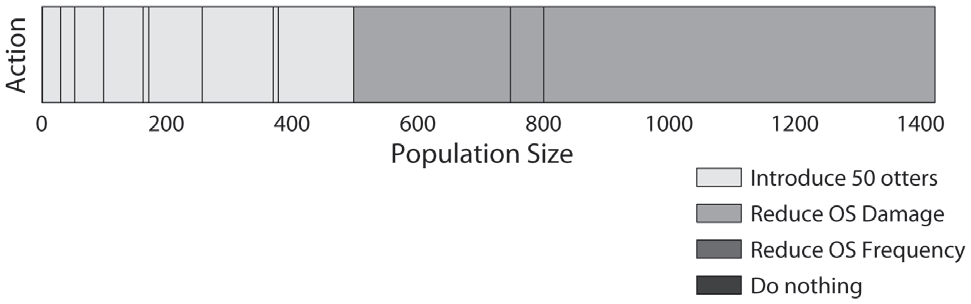
The CU-Tree state discretization showing the optimal management strategy for the fully-observable sea otter population.

Our first step in solving the sea otter management problem was to determine the optimal management strategy for the population assuming perfect detectability. As noted above, we need to keep the number of states that are used in the discretization small so that the partially observable problem will be tractable. This is achieved using the CU-Tree algorithm (see methods section). The CU-Tree algorithm compactly represents the solution for any population size using 13 states (figure 1). Re-introduction of new otters is the best option for small populations of sea otters and reducing oil spill damage is the best option for populations larger than 497 otters.

### Managing the sea otter with imperfect detection

The CU-Tree solution assumes that the observed population size is the true population size. We call this solution perfectly observable, or just the Markov decision process (MDP) solution. In reality, managers are never certain that the observed state after sampling is the true state. A population census may underestimate (e.g. the survey may not detect all individuals) or overestimate the number of individuals in the population (e.g. by double counting or inaccurate extrapolation of representative samples). We account for this uncertainty by recording the probability that we are in a state based on the previous behaviour of the population. Our optimization model becomes a partially observable Markov decision process (POMDP).

**Figure 2:**
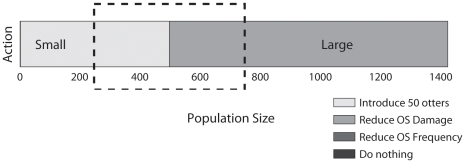
Schematic showing how observation probability is accounted for in the sea otter POMDP. Only two observations matter for management, corresponding to “small” and “large” populations. For populations that are very large or very small (outside the rectangle in the figure), the manager can observe the correct state perfectly. For the states within the rectangle (i.e. states within 250 otters of the population size where the optimal action changes), managers can only observe the correct state with probability 0.5, unless monitoring is undertaken.

The structure of the optimal solution for the perfectly-detectable population (figure 1) is such that there are only two important actions– introduce otters when the population is small, or reduce the intensity of oil spills if the population is large. This means that even though the true population cannot be counted exactly, the true population size will not affect the best management action unless the true population size is close to the point where the best management action changes. Using this information we constructed and solved a POMDP for the sea otter problem (see methods section and figure 2). Solving the POMDP means we can take the best action even if we are unsure about the true size of the population.

We found exact solutions to the finite-horizon POMDP for up to 6 timesteps. However if we need to manage the population indefinitely an infinite time horizon solution is required. This was too computationally demanding to solve using an exact algorithm, so we solved the infinite time problem using the point-based approximation algorithm Perseus [6].

**Figure 3:**
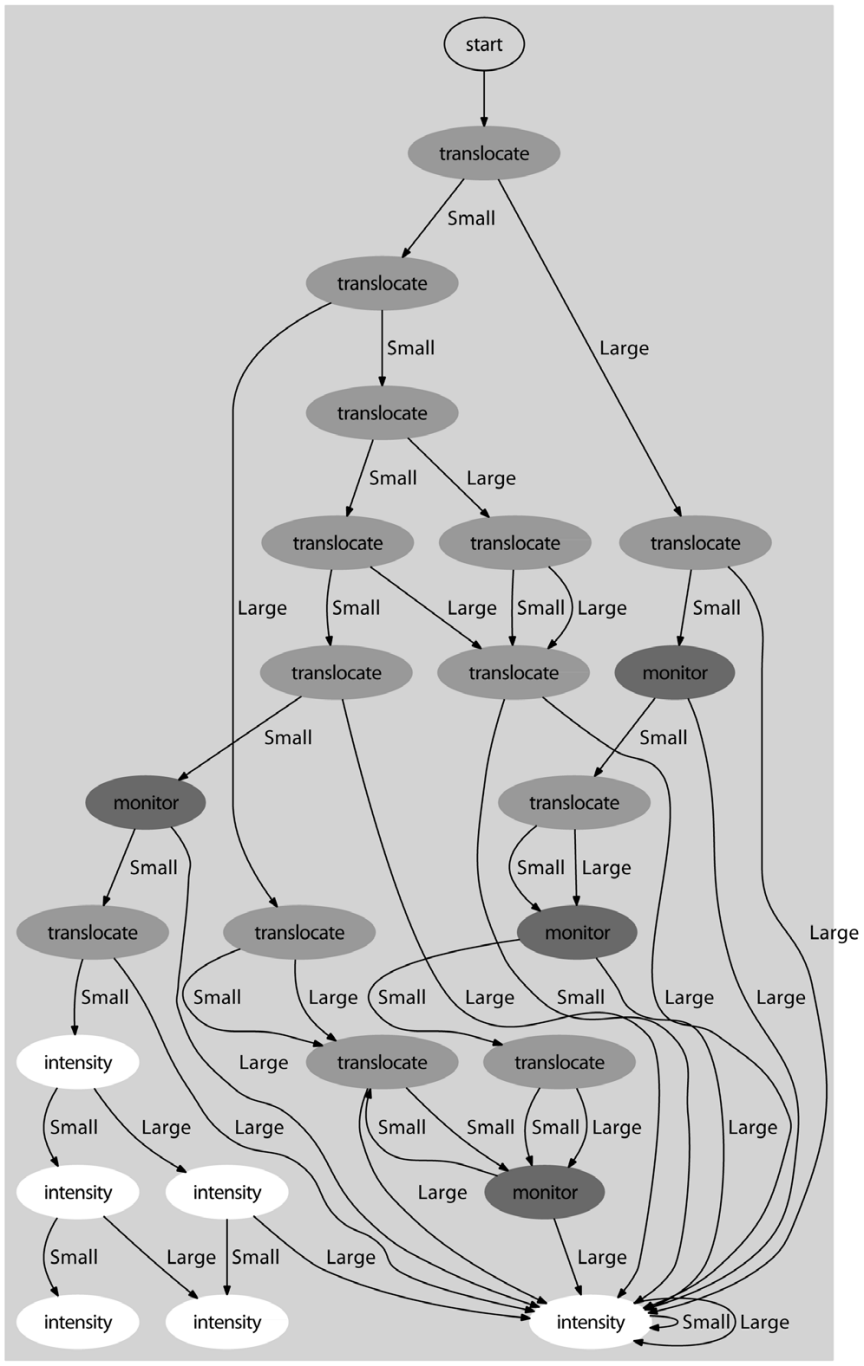
Approximate infinite-time horizon policy graph where all states have the same initial belief.

An optimal POMDP solution is presented as a policy graph. A policy graph depicts states and optimal actions as nodes of a graph. The outcomes of the possible observations are contained in the arrows connected to the node. Using the Perseus solution, we derived an approximate infinite-time horizon policy graph where all states are initially allocated equal belief (i.e. we have no prior information about the population) (figure 3). It is impractical to show the full policy graph because the solution contains 353 nodes. Figure 3 shows all the optimal observations and actions for a 10-timestep simulation. The optimal strategy is to initially avoid extinction by assuming the population is small and re-introducing otters. This is repeated unless we observe a large population sufficiently many times that we become sure that the correct observation is large, at which point the optimal action becomes to reduce the damage caused by oil spills if they occur. Monitoring is optimal when we make conflicting observations and are in a belief state where we are uncertain about which of the two actions is optimal.

### The value of information: comparing performance with and without perfect observability

**Figure 4:**
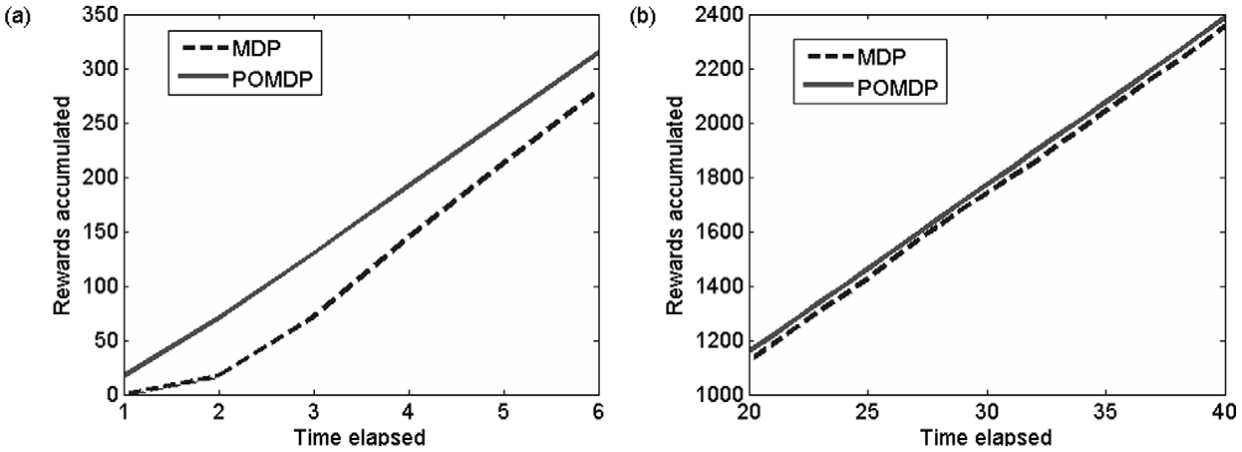
Performance plots showing rewards accumulated using both the MDP and POMDP strategies calculated over (a) a finite 6-year horizon calculated using an exact algorithm (Cassandra's algorithm); and (b) between 20 and 40 years calculated using the infinite time approximate Perseus solution. More information on Cassandra's algorithm and Perseus are included in the methods section of the manuscript.

**Figure 5:**
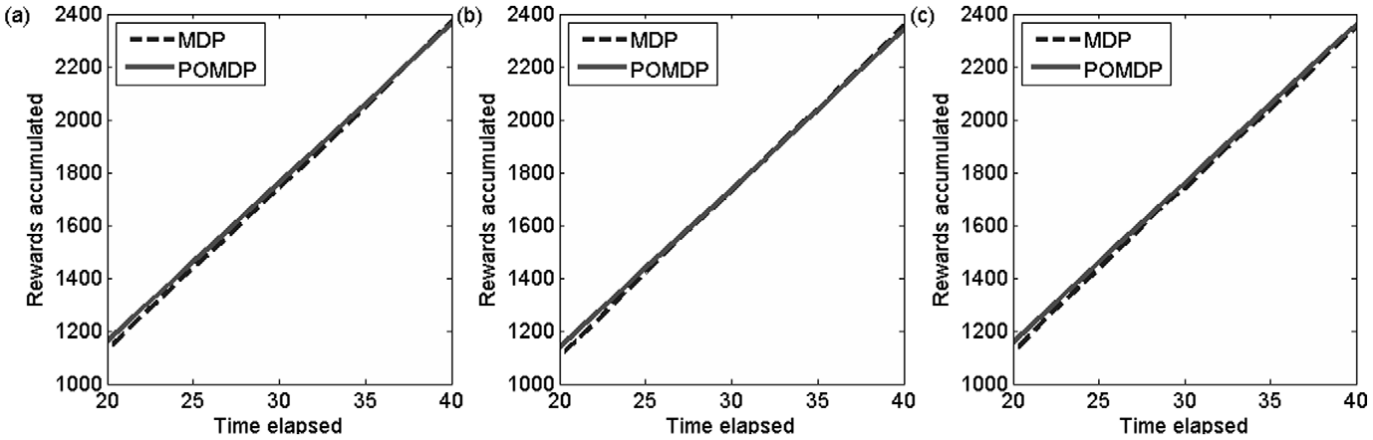
Performance plots showing rewards accumulated using both the MDP and POMDP strategies calculated with variable costs the long term (20–40 years). All figures were calculated with the infinite time approximate Perseus algorithm. Figures show: (a) cost of monitoring = 10% of management action cost; (b) cost of monitoring = 20% of management action cost; (c) cost of monitoring = 100% of management action cost. In the long term, monitoring does not make a difference to the objective regardless of the relative management cost. More information on Cassandra's algorithm and Perseus are included in the methods section of the manuscript.

To test the value of monitoring the otter population, we simulated the partially observable sea otter population and tested a perfectly observable (MDP) strategy and a POMDP strategy in which we account for our observation error. In the MDP strategy we ignore the possibility that our observation can be inaccurate (so it is never worthwhile to monitor, but it is possible to unwittingly choose suboptimal actions), but in the POMDP strategy we explictly account for observation error and allow for monitoring. Figure 4 plots the performance of the MDP and POMDP strategies on the otter problem averaged over 20 simulation runs. Initially, the POMDP outperforms the MDP because the POMDP maintains some belief that the population is in the reward state at each time step, while the MDP must explicitly visit the reward state to accumulate any reward (figure 4a) (see methods section for the definition of reward state). However in the long term the MDP and the POMDP give the same performance for this particular problem (figure 4b). This behaviour is a result of the high growth rate of the sea otter population (which causes the population to rapidly approach the carrying capacity) as well as the simple state discretization created by CU-Tree (in which all of the reward is contained in one state, and only two states are affected by poor observation). We obtained similar results for different relative values of monitoring cost (figure 5). Because the same reward is achieved in the long term regardless of whether or not we account for partial observability, investing in intensive monitoring is unnecessary in the case of the Washington sea otter. However this is not a general result for all POMDP problems. The sea otter population is robust to suboptimal MDP management but more threatened populations have been shown to benefit from being managed as a POMDP [7].

## Discussion

We have shown how discretization methods can be used to make optimal decisions for a continuous partially observable Markov decision process. By choosing states based on the effects of management actions, the number of states required to compute the optimal solution is greatly reduced.

Given that the CU-Tree solution suggests just two actions, it is tempting to reduce the state space to only two states, with one representing each action. This creates fewer states, but the values of the two states are no longer known. The minimum requirement for preserving the optimal policy is that two states that are aggregated into the same class must have the same optimal Q-value [8] (see methods section for definition of Q-value). Under this condition the aggregate states will yield a value function whose greedy policy is optimal in the original, non-aggregated MDP (i.e. the action that maximizes immediate reward in the aggregate state is the same as the actions that maximize the long-term expected sum of rewards of the original states that comprise the aggregate state). This means that we can devise aggregation techniques that alter the reward structure and transition probabilities between states, but we must preserve the mapping of Q-values to actions to maintain the same optimal management strategy. CU-Tree satisfies the conditions for maintaining the optimal actions after aggregation.

CU-Tree automatically splits the states wherever the Kolmogorov-Smirnov test finds that the sampled distributions on either side of the proposed split are significantly different. The splits do not occur in exactly the same location in every run of the CU-Tree algorithm, but depend on which states are sampled during the data gathering phase. The number of splits made by the algorithm also varies, and generally increases as the number of loops through the data gathering phase increases (we ran the algorithm for 50 loops). This means there is a trade-off between creating more splits to refine the Q-values (see methods section) and keeping the number of splits low enough to effectively solve the POMDP. We found that CU-Tree rapidly identified the population size where the actions change (496 otters) but took longer to split the other states more finely. As described previously, what matters more than the exact split locations is the management action that is recommended for each state. The states that appear as narrow bands in figure 1 are a product of this stochastic state-splitting.

Other state aggregation techniques exist that may benefit conservation. Lee and Lau [9] used an adaptive vector quantization method to partition a state space using a simulator in problems with multiple dimensions. This approach is similar to CU-Tree, however it is not clear in CU-Tree how to rank and sort the states if there are data in more than one dimension. Conservation management problems with many continuous state variables include predator-prey systems and stage-structured population models. Optimization of these large state-space problems is currently considered intractable by the majority of the conservation community. There is an opportunity to apply recent advances in continuous POMDP methods from artificial intelligence (e.g. [10], [11]) to these conservation problems.

### Using CU-Tree to manage continuous populations with imperfect detectability

Where partial observability has been accounted for in optimal management problems in conservation, it has been confined to problems with few states or very coarse discretizations (e.g. [12], [13], [14],[7]). The compact discretizations created by CU-Tree maintain the resolution of the original population and provide a missing link between the well-developed methods of population detectability in continuous models [15] and POMDP solution algorithms.

Although CU-Tree enabled us to reduce the state space down to 13 states in the sea otter population, not all populations will result in such a small state space. Even with 13 states, we were unable to solve the POMDP exactly for more than 6 timesteps, so more effective state space compaction methods would be very useful. Although approximate methods like Perseus [6] can obtain near-optimal policies for the infinite-time case, very large state spaces will reduce the accuracy of approximate methods.

Despite these issues, populations are frequently modelled with the same structure (e.g. logistic growth) and similar management objectives (i.e. grow or eliminate the population). We suspect this structure is reflected in the optimal management strategy. Because the population increases smoothly, similar population sizes will tend to require the same management action (e.g. small population sizes generally need an action that prevents extinction, whereas the action for larger populations is often to prevent catastrophic mortality). This local similarity between datapoints should result in a discretization with relatively few states. An area for future research is to investigate whether the underlying structure of population models may ensure that the number of states and observations (i.e areas where the management action change) remains relatively small.

### Lessons for sea otter management

The optimal management strategy for the sea otter with imperfect observability depends on the relative cost of monitoring compared to management. Where monitoring costs were the same as management, monitoring was never optimal. As monitoring costs decrease relative to management, monitoring becomes optimal when conflicting information is received about the true state of the otter population (figure 3). Despite these differences, monitoring did not affect long-term utility for this problem for a range of relative costs (see figure 5). For a detailed study about how management and monitoring decisions are affected by monitoring cost and belief state, see [13].

Under the assumptions of this study, imperfect detection of the sea otter population size was not important in the long term, as it gave results that were the same as if perfect detection was assumed. This long-term behaviour suggests that if a healthy population of otters can be maintained for some time, then the best course of action is to manage the population optimally despite having some uncertainty about the exact population size. Rather than expend funding on monitoring, the funding would be better used on managing the threat of oil spills on the otter population.

## Materials and Methods

### Management objective

Published estimates of the carrying capacity 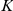 for the Washington sea otter population vary [5], [16] dependening on the modelling method used. For this study we adopt the best estimate of 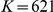 otters from the Gerber model. We assumed that a healthy population size is a population that is within 20% of the carrying capacity, so the management objective is to achieve a sea otter population between 497 and 746 individuals.

We note that this target differs from that in the Washington sea otter recovery plan [17] that is currently used to manage the population (downlist from State Endangered to State Threatened when population is maintained between 1640–2187 otters). We use the Gerber [5] estimate as it provides a population growth model, which is essential for obtaining the state-based management solution. We used a different target to the recovery plan as the Gerber model will very rarely (if ever) reach the target population size given the current parameters.

### The model

Gerber et al. [5] found that the data fit a Beverton-Holt model and logistic growth (Ricker) model equally well. In this study a modified stochastic Ricker model 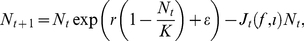
(1)


is used to model the otter population, where 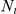 is the number of otters at time 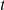, the growth rate 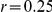 and the carrying capacity 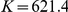 [5]. The environmental stochasticity 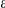 is normally distributed according to 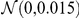. The term 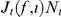 gives the otter mortality due to an oil spill. Spills occur randomly with frequency 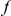 and damage 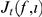, where 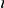 is a random draw from a uniform distribution that determines whether an oil spill occurs. If a spill occurs, the proportion of otters killed by a spill 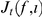 is chosen randomly between a maximum and a minimum damage.

Although the sea otter is unlikely to go extinct in the absence of oil spills, the risk posed by oil spills on the Washington coast remains uncertain [5]. For the purposes of this paper we assumed that the oil spill frequency 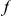 is 0.3, and oil spill mortality 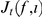 is between 0–50%. We carried out sensitivity analysis on these parameter values but found that the choice of frequency and mortality did not signficantly affect the recommended management action (see file S1).

We allow four management actions for the sea otter population. Only one action can be taken each year. We assume that a management agency can: (i) reduce the frequency of oil spills by 20%, (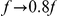 in equation 1) for example by restricting shipping near sea otter habitat; ii) reduce the damage caused by an oil spill by 20% (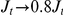); (iii) re-introduce 50 otters (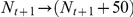 in equation 1), for example otters can be introduced from healthy populations in other parts of North America, or (iv) do nothing (no effect on model). Although translocation of 50 otters is quite a large translocation, we expect that the costs of redirecting oil tankers or oil spill cleanup will be expensive. Translocating 50 otters may become feasible when compared with these costly actions. There may also be other barriers to management implementation such as inter-agency cooperation, but these matters are not addressed here. In this manuscript we focus solely on achieving the management objective for the sea otter population.

### Optimal management of sea otters

#### State space discretization

In this section we demonstrate how to use the expected effects of management actions to choose a compact set of states that are necessary to solve the optimal decision problem in a Markov decision process framework [18]. We first assume that the population is perfectly observable, then add partial observability later in the paper. The continuous U-Tree algorithm [19] (CU-Tree), an extension of the earlier U-Tree algorithm [4], generates a state space discretization and optimal management policy from a continuous state space with discrete actions. The method finds the positions where the optimal action changes to a greater resolution than a coarse uniform discretization and does not require prior knowledge of the transition probabilities.

We use CU-Tree to learn a discretization from the population model given by equation 1. CU-Tree goes through two phases to build a discretization: a data gathering phase, in which the population model is simulated to obtain data, and an expansion phase, where the simulated data is used to create new states by splitting the existing states according to a splitting criterion. We denote the state space using 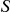 and the action space using 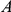. Initially, CU-Tree has just one state 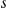, which encompasses the whole range of possible population sizes (i.e. 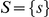).

In the data gathering phase, the model (equation 1) is used to simulate a number of trajectories of a given length (we used trajectories of 50 timesteps in this paper). All simulated data is stored as datapoints. A datapoint is defined as the quadruple 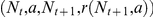, where 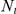 and 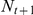 are the population sizes at times 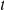 and 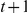 respectively, 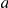 is the action taken at time 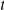, and 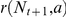 is the immediate reward associated with population size 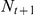 and action 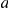. Datapoint rewards (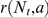) are allocated according to the current population size and management action. We allocate a reward of 100 points for all population sizes within 20% of the carrying capacity (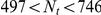) and subtract the cost of action from this reward. Management actions other than ‘do nothing’ incur a cost of 10 units each. The datapoint reward 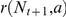 is defined as: 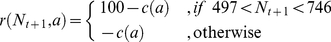
(2)


where the cost of action 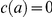 if the action is ‘do nothing’, and 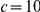 otherwise.

Estimates of the transition probability 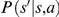, state reward function 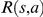 and Q-values 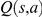 are updated at each simulation timestep. The transition probability from state 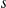 to state 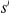 after taking action 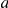 and the expected future reward function are updated according to [20]: 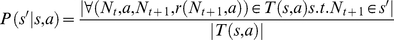
(3)

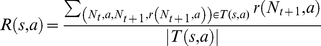
(4)


where 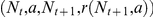 is the datapoint being updated and 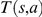 is the set of all transition tuples currently stored in the transition history that have starting state 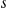 and use action 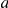.

The Q-values record the current value of each state and are updated using the Bellman equation [18], [21]: 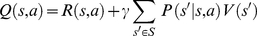
(5)


where the value function 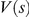 is given by 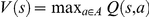. As datapoints are added to the Q-values, 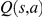 will converge to the global reward obtained by applying a management action 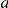 to state 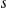.

**Figure 6:**
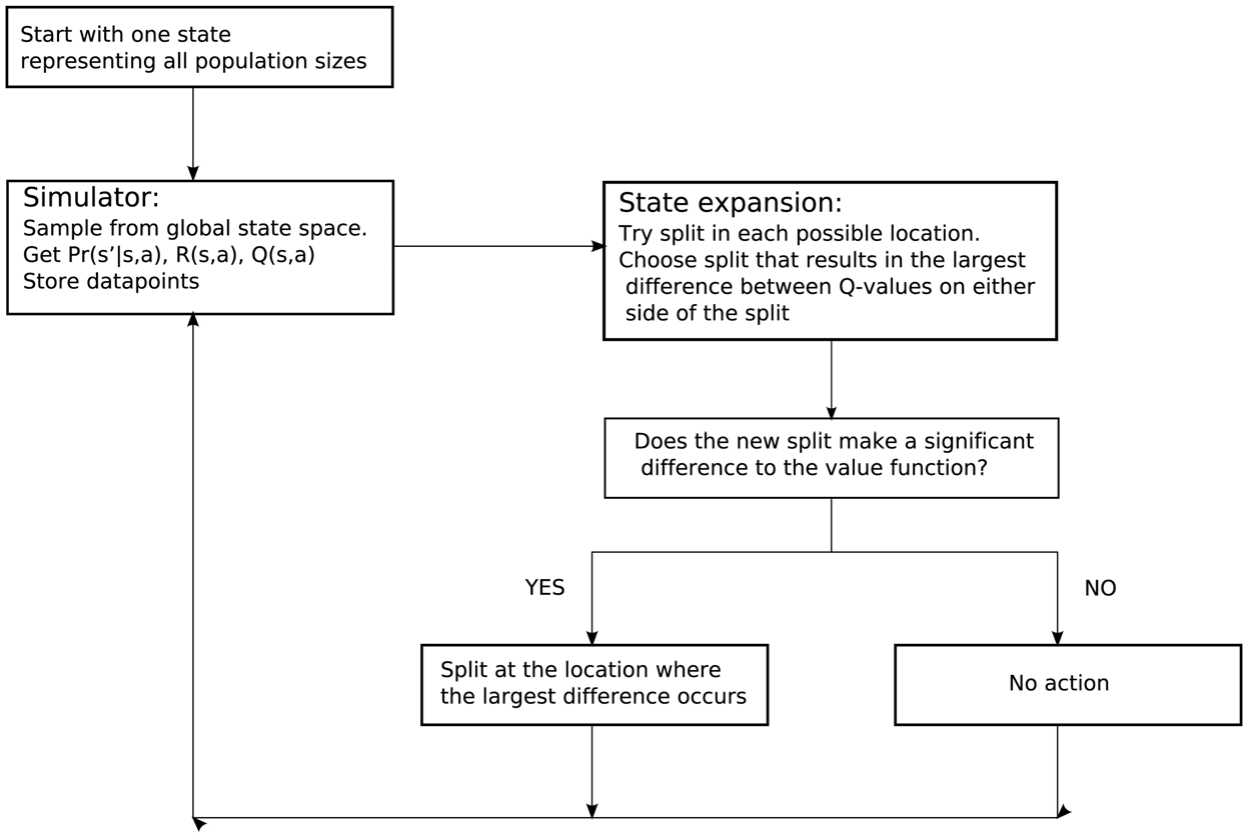
Block diagram of the continuous U-tree algorithm. Datapoints are collected and stored by simulation using the population model. For each state, datapoints are sorted into ascending population sizes. Trial splits are added between every stored datapoint and the trial split that results in the largest difference in Q-values is selected. If the difference in Q-values of the distributions on either side of the trial split is statistically significant, then a split is added and a new state is created. If the difference in Q-values is not significant, then no split is added. The algorithm is repeated until a stopping criterion is reached.

After the data collection phase, an expansion phase checks whether new states should be added. Trial splits are added between every stored datapoint and the trial split that results in the largest difference in Q-values is selected. If the difference in Q-values of the distributions on either side of the trial split is statistically significant then a split is added and a new state is created. Significance is tested using a two-sample Kolmogorov-Smirnov test [22] with a null hypothesis that the two distributions are from the same distribution (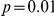). The algorithm is repeated until no significant splits can be added. The block diagram in figure 6 summarises the steps involved in CU-Tree.

CU-Tree will eventually converge to the optimal management policy. The optimal management policy is obtained from the discretization using the Q-values generated by CU-Tree. The optimal policy 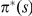 for state 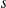 is the action that maximizes the value function: 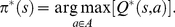
(6)


where 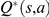 are the Q-values 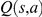 after many iterations of the algorithm. The sampling loop was repeated 250 times to generate the discretization for the sea otter population. A MATLAB implementation of CU-Tree applied to the sea otter problem is included in file S2.

#### Partially observable Markov decision processes

The CU-Tree solution assumes that the observed population size is the true population size. We call this solution perfectly observable, or just the Markov decision process (MDP) solution. In reality, the problem is partially observable because managers cannot detect all the individuals in the population in a census. A partially observable Markov decision process (POMDP) consists of a discrete state space 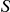, a discrete set of actions 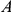 which can be applied to 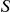, and immediate rewards 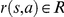 that will be received for taking action 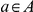 on state 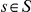. The environment changes from state 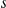 to state 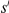 according to a transition probability model 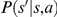. Because the problem is partially observable, a manager of the system cannot know the state of the system exactly but will instead make observations 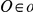 that give information about the true state of the system through an observation function 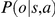. As observation data is collected, the manager builds up a belief state 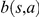 that gives the probability that the state observed after taking an action is the true population state. Interested readers can refer to [23] for further information on POMDPs.

The CU-Tree discretization for the sea otter problem reduced the state space to 13 states, and we use this to define the state space for the POMDP (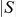 = 13). The objective of the POMDP is to discover the optimal action to take given a set of beliefs that we are in each state– in this case to find the actions that will maintain the sea otter population within 20% of the population carrying capacity with the highest probability. We allow the same four actions as the perfectly observable case, as well as an option to monitor the population (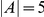 actions). Monitoring the population temporarily increases the probability that we observe the correct state. We assume that all management actions cost 10 units, but monitoring costs 2 units. Transition probabilities and rewards are derived from CU-Tree (equations 3 and 4).

To implement a POMDP we define the possible observations 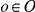 and the probabilities that our observations are correct 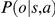. CU-Tree defined 13 states for the sea otter population (see results section). The optimal actions for the first 10 states are identical, and the optimal actions for the remaining 3 states recommend a second action. This means that a manager must choose between two actions based on his belief of the current system state. For this reason we allow just two observations – a manager will either observe a ‘small’ (0–496 otters) population, or a ‘large’ (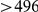 otters) population and act accordingly. Note that by choosing just two observation states we have not reduced the number of states in the problem. We cannot reduce the number of states to less than the number of states in the CU-Tree discretization because this will mean that we change the problem we are solving and lose our estimates of the transition probabilities and rewards for each state [8].

We assumed that managers always observe correctly in all states except for those near where the optimal decision changes. We set the probability 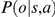 of observing correctly to be 1 for all states that occur outside of a ‘window’ of width 250 otters around the point where the optimal decision changes (around the point where the observation changes from ‘small’ to ‘large’) (figure 2). For states that fall within this width, managers may observe incorrectly – we set the probability of making the correct observation from these states to be 0.5. If the action ‘monitor the population’ is chosen, the probability of making the correct observation in these states rises to 0.95.

We solve a partially observable Markov decision process (POMDP) by maximising the expected future rewards over time. The reward in a POMDP is computed as the expected reward across all belief states. Future rewards must account for whether the observed state is the correct state. The POMDP was solved with Cassandra's pomdp-solve software [24] for up to 6 timesteps. Solving this problem exactly for more than 6 timesteps was too computationally expensive. The point-based approximation algorithm Perseus [6] was used to find an approximate infinite time solution. Details of POMDP solution methods can be found in [23] and [6].

## Supporting Information

File S1 **Sea otter management: a sensitivity analysis.**
(PDF)

File S2 **MATLAB M-files to implement CU-Tree for the sea otter problem.**
(TAR)
