# Supplementary material for: Which States Matter? An Application of an Intelligent Discretization Method to Solve a Continuous POMDP in Conservation Biology
Source: PLoS One. 2012 Feb 17;7(2):e28993. doi: 10.1371/journal.pone.0028993 (PMC3281817; doi:10.1371/journal.pone.0028993)
Supplement: File S1 — Sea otter management: a sensitivity analysis. (PDF) [file pone.0028993.s001.pdf]

Appendix 1: Sea otter management: a sensitivity analysis

Following from [1], sensitivity analysis was performed for oil spills of different intensities and frequencies (figure 1). In all scenarios tested, the optimal action was to translocate otters when the population is small, and expend effort on reducing oil spill intensities if they occur when the population is large. In the case where the frequency and intensity of oil spills is small, there are a number of states where the optimal action is to do nothing rather than to attempt to decrease the oil spill intensity. Reducing the oil spill intensity by 20% makes comparitively little difference for these population sizes, and the cheaper cost of doing nothing means that decreasing intensity is not optimal for these states. Attempting to reduce the frequency of oil spills was not a consistently optimal action in any of the scenarios tested. As the predicted intensity of the oil spills increases, the point at which the optimal management action changes from translocation to reducing oil spill intensity decreases (for example for an oil spill frequency of 0.3, the split point was located at 440, 340, 320 and 220 otters for intensities of 0-50%, 25-50%, 25-75%, and 50-75% respectively). Increasing the oil spill frequency did not have noticable effect on the split point.

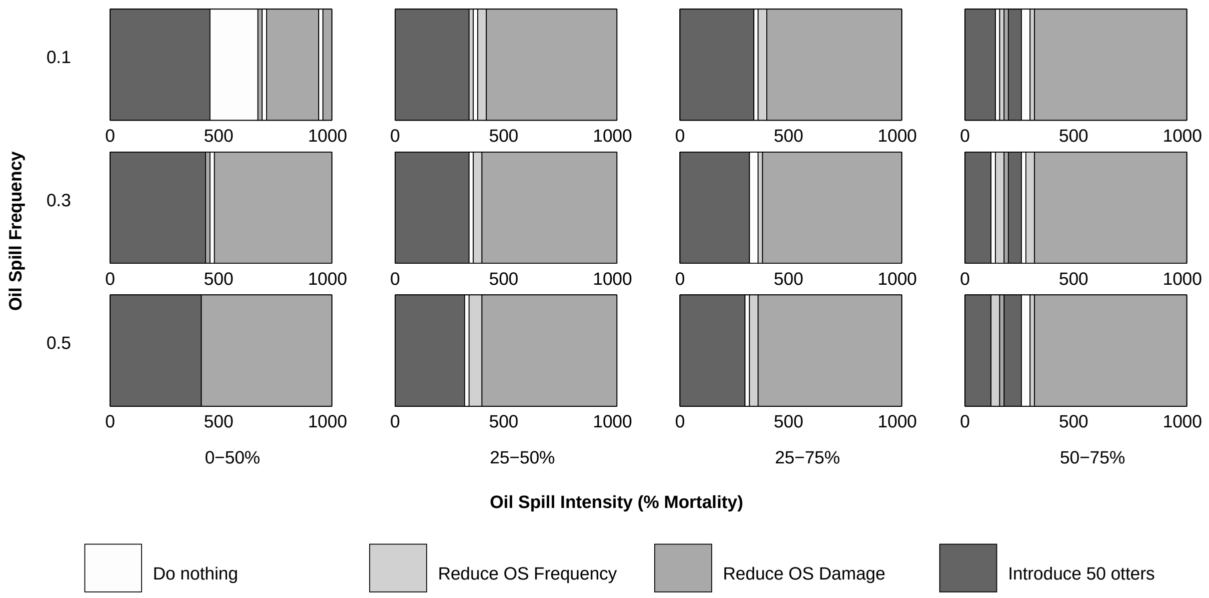

Figure 1. Optimal management strategies for the Washington sea otter for different oil spill frequencies and intensity ranges. The abbreviation OS represents oil spill.

References

1. Gerber LR, Buenau K, Vanblaricom G (2004) Density dependence and risk of extinction in a small population of sea otters. Biodiversity and Conservation 13: 2741-2757.
